# Biodistribution and Biodegradation of an Osteoinductive Supramolecular Polymer Implant in a Rat Spinal Fusion Model

**DOI:** 10.3390/jfb17030107

**Published:** 2026-02-24

**Authors:** Jacqueline Inglis, Alyssa Goodwin, Steven Kurapaty, David M. Hiltzik, Rahim Laiwalla, Hogan Brecount, Nicholas A. Sather, Emily A. Waters, Chad R. Haney, Rebecca Sponenburg, Xinyi Lin, Wellington K. Hsu, Samuel I. Stupp, Erin L. Hsu, Romie F. Gibly

**Affiliations:** 1 Department of Orthopaedic Surgery, Feinberg School of Medicine, Northwestern University, Chicago, IL 60611, USA; 2 Center for Regenerative Nanomedicine (CRN), Northwestern University, 303 E. Superior, Chicago, IL 60611, USA; 3 Center for Advanced Molecular Imaging in the Chemistry of Life Processes Institute and Biomedical Engineering, Northwestern University, Evanston, IL 60208, USA; 4 Department of Biomedical Engineering, Northwestern University, Evanston, IL 60208, USA; 5 Department of Radiology, Northwestern University, Chicago, IL 60611, USA; 6 Department of Chemistry, Northwestern University, Evanston, IL 60208, USA; 7 Department of Biomedical Engineering, Northwestern University, Evanston, IL 60208, USA; 8 Department of Materials Science and Engineering, Northwestern University, Evanston, IL 60208, USA; 9 Department of Medicine, Northwestern University, Chicago, IL 60611, USA; 10 Division of Orthopaedic Surgery and Sports Medicine, Ann & Robert H. Lurie Children’s Hospital of Chicago, Chicago, IL 60611, USA

**Keywords:** bone graft substitute, spinal fusion, spine surgery, supramolecular polymer, peptide amphiphile, biodegradation, biodistribution, osteoinductive

## Abstract

Recombinant human bone morphogenic protein-2 (rhBMP-2) use in spinal fusion is limited by dose-dependent complications. Peptide amphiphile (PA) supramolecular polymers presenting a BMP-2–binding epitope have previously been developed to reduce the rhBMP-2 dose required for successful fusion. We evaluated PA implant biodegradation and tissue clearance in a rat posterolateral spinal fusion model as a prerequisite to clinical safety studies. Twenty-three female Sprague–Dawley rats underwent L4–L5 fusion with gadolinium (Gd)-labeled PA implants. Longitudinal magnetic resonance imaging (MRI) was performed up to 13 weeks postoperatively, while the spine and filter organs were harvested for inductively coupled plasma mass spectrometry (ICP-MS) quantification of Gd at multiple time points. Gd concentration at the fusion site decreased from 71% of maximum to 19.5% at 13 weeks, and MRI showed a complete loss of Gd signal enhancement by 8 weeks. In peripheral organs, peak Gd accumulation was 3% in the liver at 4 weeks, declining to 1.4% at 13 weeks, while Gd remained below 0.05% in the spleen, lung, and blood at all time points. These data indicate PA implant localization, with robust degradation and clearance and minimal off-target accumulation, supporting its translational potential for spinal fusion applications.

## 1. Introduction

Spine fusions are common orthopedic procedures for disc degeneration as well as spinal instability and deformities, and frequently these procedures require a bone graft material to achieve fusion. Since over 500,000 fusions are performed annually [1], an effective graft that promotes fusion and healing is essential. Historically, iliac crest bone grafting (ICBG) was the gold standard, but this procedure now comprises less than 6% of spinal fusions due to donor-site morbidities, extended lengths of surgery and hospital stays, higher blood loss, greater rates of infection, and acute and chronic pain [2,3]. Nonunion rates following posterior spinal fusion have been reported to be between 8.1% and 43.3%, and costs associated with reoperations for symptomatic pseudoarthrosis have been reported to be between 24,000 and 64,000 dollars per patient [4,5]. Consequently, there is a need for alternative graft materials conducive to consistent bone growth while minimizing complications.

Recombinant human bone morphogenetic protein-2 (rhBMP-2) was FDA-approved for single-level anterior lumbar interbody fusion (ALIF) in 2002. It showed great success with high fusion rates, and its indications were expanded throughout the early 2000s, with dramatically increased clinical utilization [6]. As rhBMP-2 use increased, the side effect profile became better understood. Major complications include radiculopathy, ectopic bone formation, infection, hematoma, urogenital complications, and inflammation. In the cervical spine, soft-tissue inflammation has been linked to airway compromise and breathing emergencies [7,8,9,10]. While rhBMP-2 was initially thought to improve upon ICBG, the risk profile presented in later studies highlighted the need for safer yet efficacious bone graft substitutes.

In 2001, Hartgerink et al. pioneered the development of peptide amphiphile (PA) supramolecular polymers; nanostructured scaffolds that can lead to biomimetic hydroxyapatite mineralization, enabling a natural orientation of crystals with respect to collagen fibers in bone [11,12]. PAs are composed of peptides that are designed to self-assemble into nanoscale filaments and can be further crosslinked in physiologic fluids to form hydrogels that can localize bioactive signals [13,14,15]. PA hydrogels form networks architecturally analogous to natural extracellular matrices [11,12,16]. This discovery created an opportunity to incorporate a new class of materials into spine procedures that could enhance mineralization using bioactive signals and the biomimetic structure of PA hydrogels.

To reduce the side effects associated with high rhBMP-2 doses, we developed a PA with a BMP-2-binding epitope (BMP-2-binding PA) to promote the localization of both endogenous and recombinant BMP-2, potentially reducing the therapeutic dose needed for fusion [17]. We recently reported the development of a BMP-2-binding PA formulation that incorporates collagen microparticles to form a supramolecular polymer slurry (SPS) that is injectable or moldable for placement into bony implant sites. This material showed significant potential in small and large animal models, eliciting robust spinal fusion rates using rhBMP-2 doses 100-fold lower than levels traditionally required, due to the controlled release of growth factor from the BMP-2-binding supramolecular polymers [18,19].

The PAs within the SPS are composed of peptides and fatty acids that are designed to be fully biodegradable. In preparation for human trials, it is important to quantify the PA degradation rate and evaluate the elimination pathways. Prior studies have successfully labeled PA with gadolinium (Gd) and utilized magnetic resonance imaging (MRI) to evaluate the scaffold structure [20,21]. In a mouse muscle implantation study, Gd-labeled PA degradation was tracked and quantified using MRI and inductively coupled plasma mass spectroscopy (ICP-MS). Gd MRI signal enhancement was absent at day 15, and ICP-MS showed approximately 30% Gd remaining in the muscle at 24 days post-implantation, highlighting the sensitivity differences between the MRI and ICP-MS [22]. Note that MRI does not image Gd directly; Gd changes the local environment causing MRI signal enhancement while ICP-MS directly measures Gd content. For spine fusion applications, the bone-healing environment differs in important ways from muscle, requiring further investigation. The present study aims to use Gd-labeled BMP-2-binding PAs in conjunction with live-animal MRI and ICP-MS to quantify and track PA breakdown following implantation in the spinal fusion setting. This will provide important data regarding the biodistribution of the degrading material that must be considered for the translation of this promising technology for human use.

## 2. Materials and Methods

### 2.1. Peptide Amphiphile Synthesis

PA molecules were synthesized with the following sequences: C_16_-VVVAAAEEE (diluent PA), TSPHVPYGGGS-EEEAAAVVV(K)-C_12_ (BMP-2-binding PA), and C_16_-VVVAAAEEE-K (dodecane tetraacetic acid (DOTA)-Gd) (Gd PA). Diluent and BMP-2-binding PA molecules were prepared using standard 9-fluorenyl methoxycarbonyl (Fmoc) solid-phase peptide synthesis and purified by reverse-phase high-performance liquid chromatography (HPLC) in a water–acetonitrile gradient, as described previously [19]. The Gd PA was first synthesized as C_16_-VVVAAAEEE-K(Mtt). The methyltrityl (Mtt) protecting group was then removed from the lysine while still on resin using 3% trifluoroacetate (TFA) in dichloromethane (DCM) with 5% triisopropylsilane (TIS). After washing with DCM and dimethylformamide (DMF), DOTA-tris(tert-butyl ester) (Tri-tert-butyl 1,4,7,10-tetraazacyclododecane-1,4,7,10-tetraacetate) was added using 1.5 equivalents, with 8 equivalents diisopropylethylamine (DIEA) and 1.5 equiv. (Benzotriazol-1-yloxy)tripyrrolidinophosphonium hexafluorophosphate (PyBOP) in DMF for 18 h at room temperature. The peptide was then cleaved from resin using 95% trifluoroacetic acid (TFA), 2.5% water, and 2.5% TIS, precipitated with cold ether and purified by HPLC. Pooled fractions containing C_16_-VVVAAAEEE-K(DOTA) peptide were frozen and lyophilized to dryness. Purified DOTA peptide was then dissolved in Milli-Q water, 1.5 equivalents of GdCl_3_ were added and the pH was adjusted to 6. The solution was allowed to stir at room temperature for 18 h, and Gd chelation was confirmed by mass spectrometry. The Gd PA was then purified by HPLC and lyophilized to dryness. The absence of free Gd in the final Gd PA product was confirmed using a xylenol orange test [23]. All PAs were confirmed to be at least 95% pure by liquid chromatography-mass spectroscopy (LC-MS) and stored at −20 °C until use (Figure S1).

### 2.2. Implant Preparation

PA solutions were prepared at 11 mg/mL (63 mol% diluent PA, 32 mol% BMP-2-binding PA, and 5 mol% Gd PA) by dissolving in Milli-Q water, adjusting to pH 7.5 using 1 M NaOH, and then annealing at 80 °C for 30 min and slow-cooling to room temperature overnight. On the day of surgery, a 1.5 mg/mL rhBMP-2 solution (Infuse, Medtronic, Memphis, TN, USA) was diluted in milli-Q water to 3.8 µg/mL before mixing in a 1:9 ratio with the PA solution, resulting in a PA/BMP-2 solution with a final PA concentration of 10 mg/mL (1 wt%) and a rhBMP-2 concentration of 50 ng per 130 µL (volume per one implant).

SPS implants were then prepared according to the previously described method [19]. Briefly, absorbable collagen sponges (Helistat, Integra LifeSciences, Princeton, NJ, USA) were homogenized in a blender and lyophilized to obtain porous collagen particles. Each SPS implant was prepared in an individual Eppendorf tube on the day of surgery by hydrating 6.5 mg of collagen particles with 130 µL of the PA/BMP-2 solution and mixing with a spatula. The implants were stored at room temperature until implantation.

### 2.3. Transmission Electron Microscopy (TEM)

PA samples were prepared for TEM by adding 10 µL of the PA solution to the shiny side of a glow-discharged grid (300-mesh copper with carbon film, Electron Microscopy Sciences, Hatfield, PA, USA) and left for 30 s. The grid was rinsed three times with Milli-Q water, stained with filtered 2 wt% uranyl acetate for 45 s, and air-dried. TEM imaging was carried out using a JEOL 1230 microscope (JEOL Ltd., Tokyo, Japan).

### 2.4. Surgical Procedure

The study was approved by and conducted in accordance with Northwestern University Institutional Animal Care and Use Committee policies and procedures under the Animal Welfare Assurance number D16-00182 and protocol IS00020440. Twenty-three female Sprague–Dawley rats (Charles River Laboratories, Wilmington, MA, USA) at ages of 12–16 weeks were utilized for L4–L5 posterolateral spinal fusion. Animals were housed in an AAALAC-accredited facility at Northwestern University (Chicago, IL, USA) in ventilated cages with ad libitum access to water and food. The holding room was maintained under a 12 h light/dark cycle with controlled temperature and humidity, in accordance with the Guide for the Care and Use of Laboratory Animals. During the procedure, rats were maintained on a heating pad under continuous anesthesia with an isoflurane inhalational anesthetic delivery system. They were monitored by an assistant for cardiac and respiratory difficulties throughout the procedure. The L4 and L5 transverse processes were exposed following a posterior midline skin incision and bilateral fascial incisions. The site was then irrigated with sterile 1 mg/mL gentamicin solution. Next, the superficial cortical layer of the transverse processes was decorticated with a high-speed burr, and the SPS implants were placed under the paraspinal musculature bilaterally, each bridging two transverse processes [18,19]. Afterwards, the fascia was closed with 3-0 absorbable sutures, and the skin incision was closed with 5-0 nylon sutures. Rats were placed in Elizabethan collars to prevent over-grooming of the surgical site and housed in separate cages. Rats were given daily collar breaks until their skin sutures were removed two weeks post-operatively. Rats were monitored weekly for health status.

### 2.5. Animal Imaging

Time points for animal imaging and tissue harvest are summarized in Table 1. For the 0-day (4 h) ex vivo time point, 3 rats were euthanized 4 h post-operatively, imaged, and harvested for tissue collection. Three rats were imaged longitudinally with MRI at all time points (2 days, and 1, 2, 4, and 8 weeks after surgery) and euthanized 10 weeks post-operatively. At each time point, 3 additional rats were imaged with MRI and then euthanized in the magnet for additional ex vivo scans followed immediately by tissue collection. One additional rat was imaged longitudinally out to 13 weeks. All longitudinal rats underwent X-ray micro-computed tomography (microCT) to confirm successful fusion.

In vivo MR imaging: Imaging was performed on a 9.4 T Bruker Biospec 9430 (Bruker Corporation, Billerica, MA, USA) with a 30 cm bore and 12 cm gradient insert, running Paravision (version 6.0.1, Bruker). The RF coil was a 72 mm quadrature volume coil (Bruker) operating in transmit/receive mode. The rat was positioned with the lumbar spine centered in the coil. Three sets of anatomical images were acquired in vivo: a T1-weighted image using an accelerated spin echo sequence (T1-RARE), a T1-weighted image using a Fast Low Angle Shot (FLASH) gradient echo sequence, and a T2-weighted image using an accelerated spin echo sequence (T2-RARE). All images were acquired with fat suppression enabled and matched slices as follows: coronal slice orientation, 50 mm × 50 mm field of view, 15 contiguous slices of 1 mm thickness. Other scan parameters are summarized in Supplemental Table S1.

Ex vivo MR imaging: At the conclusion of in vivo scanning, animals were either removed from the magnet and recovered or euthanized using either intravenous injection of sodium pentobarbital or isoflurane overdose and returned to the center of the magnet for ex vivo scanning to obtain T1 and T2 maps in the absence of animal movement and breathing. These maps were acquired using a variable-TR accelerated spin echo sequence (rareVTR) and a multi-slice multi-echo sequence (MSME), respectively. Both maps were acquired with fat suppression disabled and matched slices as follows: axial slice orientation centered on the implant, 50 mm × 50 mm field of view, matrix 256 × 192, 3 contiguous slices of 1 mm thickness. Other T1 map parameters are as follows: TR = 200 ms, 400 ms, 800 ms, 1500 ms, 3000 ms, and 5500 ms; TE = 12.7 ms; RARE factor 4; and 2 signal averages. Other T2 map parameters included TR = 4000 ms, minimum TE = 10 ms, echo spacing = 10 ms, 30 echoes, and 1 signal average.

Image processing: Anatomic MRI data were exported from the scanner in DICOM format and imported into FIJI [24] for cropping, intensity scaling, and export. T1 and T2 maps were exported from the scanner in the Bruker native format, converted into NIfTI files using JIM 7 (Xinapse Systems Ltd., West Bergholt, Essex, UK), and fit using the built-in nonlinear curve-fitting module.

### 2.6. MicroCT Analysis and Assessment of Fusion

At the 10-week time point, 2 additional spines were harvested en bloc and fixed in formalin. They remained in formalin for one week and thereafter were placed in 70% ethanol. The 10-week spines underwent blinded manual palpation by three independent observers for assessment of fusion using an established scoring system [18]. After manual palpation, the spines underwent microCT for confirmation of fusion and visualization of the fusion masses as described above. Only 2 specimens from the 10-week time point were reserved for the purpose of confirming that scaffold bioactivity is retained when Gd tags are added. The remainder of the specimens were used for ICP-MS, which precluded assessment of fusion. One additional rat was imaged 13 weeks post-operatively using a preclinical micro-positron emission tomography (microPET)/CT imaging system (Mediso-USA, Boston, Arlington, VA, USA) at 2.18 magnification, 33 µm-diameter focal spot, 1 × 1 binning, and 70 kVP, with 720 projection views over a full circle and a 90 ms exposure time. Projection data were reconstructed with a voxel size of 34 µm using a Butterworth filter back-projection software (Version 2.01, Mediso-USA). MicroCT data were exported from the scanner in DICOM format, imported into Amira 2022.1 software (Thermo Fisher Scientific, Waltham, MA, USA), filtered using a non-local means filter, and volume rendered [24].

### 2.7. Inductively Coupled Plasma Mass Spectroscopy (ICP-MS)

ICP-MS was utilized to identify Gd concentration in the spine and in filtrating organs. Tail vein blood samples were also taken from each animal prior to imaging and euthanasia. Organs were harvested into metal-free tubes in the following order: lungs, liver, spleen, kidney, and spine. Instruments were cleaned with ethanol between each tissue type to avoid cross-contamination. Tissues were digested in 2 mL trace grade nitric acid (>69%, Thermo Fisher Scientific) per gram of tissue and 0.5 mL trace grade hydrogen peroxide (>30%, GFS Chemicals, Columbus, OH, USA) per gram of tissue and incubated at 65 °C for at least 4 h to allow for complete sample digestion. Large organs (livers and spines, typically over 8 g) were first digested for at least 16 h at room temperature before heating due to the large amount of NOx gas produced. Ultrapure H_2_O (18.2 MΩ∙cm) was then added to produce a final solution of 5.0% nitric acid (*v*/*v*). Quantification of Gd in animal tissues was accomplished using ICP-MS of acid-digested samples. Gd elemental quantitative standards were prepared at two concentrations (50 ng/g and 5 ng/g Gd in 5.0% nitric acid), with serial dilutions using a Gd elemental standard (Inorganic Ventures, Christiansburg, VA, USA). A solution of 5.0% nitric acid (*v*/*v*) was used as the calibration blank.

ICP-MS was performed on a computer-controlled (QTEGRA software version 2.10, Thermo Fisher Scientific, Bremen, Germany,) Thermo iCapQ ICP-MS (Thermo Fisher Scientific) operating in STD mode and equipped with an ESI SC-2DX prepFAST autosampler (Elemental Scientific Inc, Omaha, NE, USA). Online dilution was also carried out by the prepFAST system and used to generate a calibration curve consisting of 25, 10, 5, 2.5, 1, 0.5, 0.25, 0.01, 0.005, 0.0025 ppb Gd. Each sample was acquired using 1 survey run (10 sweeps) and 3 main (peak jumping) runs (40 sweeps). The isotopes selected for analysis were 156, 157Gd, 115Indium, and 159Terbium (chosen as internal standards for data interpolation and machine stability).

### 2.8. Statistical Analysis

The ICP-MS data on Gd content were reported as the percent remaining relative to the pre-implant concentration of Gd (100%), which was determined by averaging the Gd content of three scaffolds prior to implantation. The average Gd and standard deviation were calculated using the explanted organs from each time point. Average Gd was calculated for each organ by time point (*n* = 3). The percent decay of Gd for the seven time points was calculated as follows: Gd concentration of the organ at time point/Gd concentration in the pre-implant scaffold. For each organ, the mean decay percentage at each time point was compared to the baseline Gd percentage at 4 h using ordinary, one-way Analysis of Variance (ANOVA) followed by Dunnett’s post hoc test for multiple comparisons. Statistical significance was assessed at a 95% confidence level, and power = 0.80.

### 2.9. Histology

The two spines from the 10-week time point used for microCT and manual palpation analysis were subsequently processed for histological assessment. These spines were harvested en bloc and fixed in 10% formalin at room temperature for 7 days. They were then stored in 70% ethanol until needed for histology. The spines were then decalcified in Immunocal Formic Acid Bone Decalcifier (StatLab, McKinney, TX, USA) for 49 h. The samples were then processed and embedded in paraffin. Next, 5-um-thin sections were cut with an RM2255 microtome (Leica, Nussloch, Germany). Sections were stained with a Masson’s trichrome staining kit (Sigma Aldrich, St. Louis, MO, USA).

## 3. Results

### 3.1. Co-Assembly of Nanofibers Displaying Gd and BMP-2-Binding Epitopes

**Figure 1:**
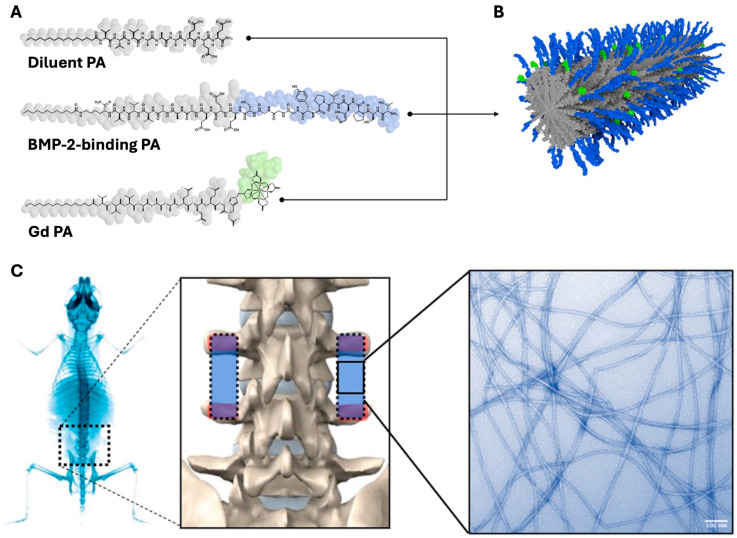
(**A**) Chemical structure of PA molecules. (**B**) Co-assembled nanostructure composed of diluent, BMP-2-binding, and Gd PA molecules, displaying the BMP-2-binding epitope (blue) and Gd complex (green) on the nanofiber surface. (**C**) Implant positioning (**left**) and false-colored TEM micrograph (**right**) of co-assembled nanofibers.

TEM imaging of PA solutions consisting of the diluent PA, BMP-2-binding PA, and Gd PA revealed nanofibrous structures (Figure 1) similar to the diluent + BMP-2-binding PA nanofibers reported previously [19]. This suggests that the co-assembly of a third PA molecule displaying the Gd epitope does not disrupt nanofiber formation. Indeed, we did not discern any differences in appearance or gross physical properties compared to the BMP-2-binding PA nanofiber solutions prepared in previous studies.

### 3.2. MRI Analysis

**Figure 2:**
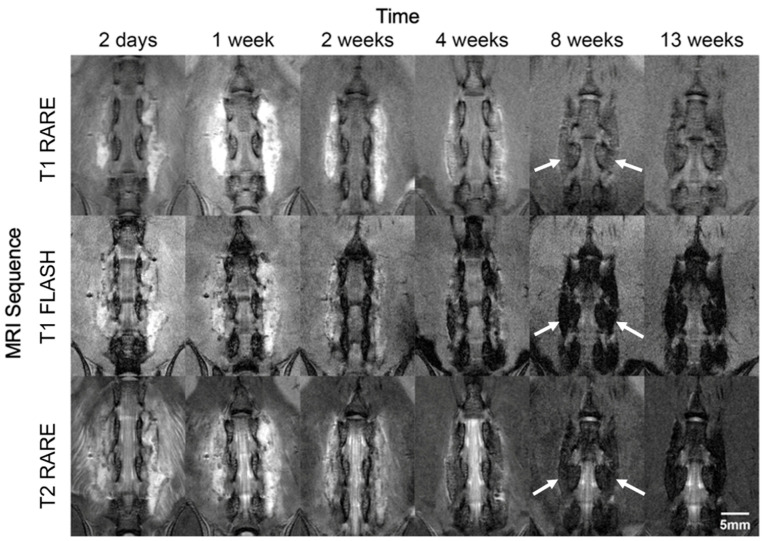
Representative in vivo coronal MR images of the spine and implant from one animal imaged over six time points (2 days, and 1, 2, 4, 8, and 13 weeks) after implantation. (**Top row**): T1-weighted RARE images showing a gradual reduction in Gd enhancement near the implant over time. (**Middle row**): T1-weighted FLASH images to confirm that the bright signal observed in the T1-RARE images was due to Gd rather than T2 bleed-through. (**Bottom row**): T2-weighted RARE images showing postsurgical edema in the paraspinal muscles that is resolved by 4 weeks. The arrows at the 8-week time point highlight a fusion mass suggestive of successfully bridging bone between L4 and L5.

Representative longitudinal MR images from one SPS-treated animal are shown in Figure 2. The T1-RARE and FLASH images showed a strong Gd enhancement in the implant through the 1-week time point. Progressive reduction in Gd enhancement was observed at the 2-week and 4-week time points, and the Gd enhancement disappeared by 8 weeks. T2-RARE images showed postsurgical edema around the implant persisting for the first 2 weeks and resolved by the 4-week time point. A fusion mass suggestive of successfully bridging bone across L4–L5 is observable at 8 weeks, as demonstrated by the homogenic features of the bridging mass highlighted by the arrows in Figure 2. T1-RARE and FLASH images were both obtained due to concerns that the RARE images could be prone to T2 effects from postsurgical edema and that the FLASH images could be prone to susceptibility artifacts at early time points due to small postsurgical air pockets and later due to bone formation. In combination, however, the two sets of images provide strong in vivo evidence of gradually decreasing Gd enhancement over time in the same animal.

Axial T1 and T2 maps in animals euthanized at each time point corroborate the in vivo longitudinal data, in that Gd enhancement was observed in the implant beginning immediately after surgery and lasting through 4 weeks post-implantation (Figure S2). Postsurgical inflammation was not observed in the animals euthanized immediately after surgery or beyond 4 weeks but was present diffusely through the paraspinal muscles between 2 days and 2 weeks.

### 3.3. MicroCT and Assessment of Fusion

**Figure 3:**
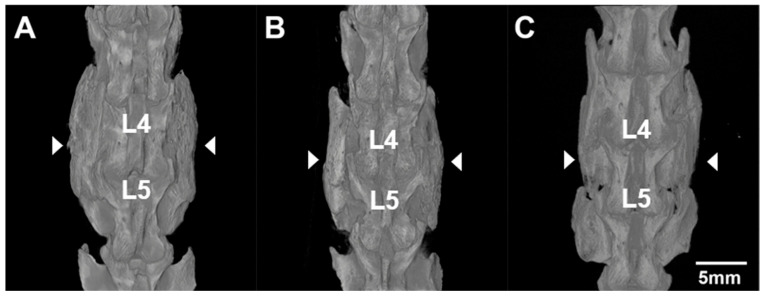
MicroCT 3D renderings of spines harvested 10 weeks (**A**,**B**) and 13 weeks (**C**) post-operatively. The L4 and L5 vertebral bodies are labeled. Arrowheads indicate areas of new bone formation and successful fusion masses bilaterally. All volumes were rendered on a grayscale range of 400–7000 HU.

Three-dimensional reconstructions from microCT imaging showed bone growth at 10 and 13 weeks post-surgery, which was indicative of successful bridging of newly formed bone across L4–L5 (Figure 3). The two spines harvested at 10 weeks both received a fusion score of 2 by manual palpation, indicating robust bilateral fusion. The 13-week spine underwent ICP-MS and was therefore unavailable for manual palpation-based fusion scoring. After microCT and palpation data were obtained, histology was performed to further assess fusion of the L4–L5 posterolateral fusion mass between the transverse processes (Figure S3). Histological slices showed a robust bony fusion mass with newly formed trabeculae and no indication of remaining SPS scaffold at the implant site.

### 3.4. ICP-MS Quantification of Gd Biodistribution

**Figure 4:**
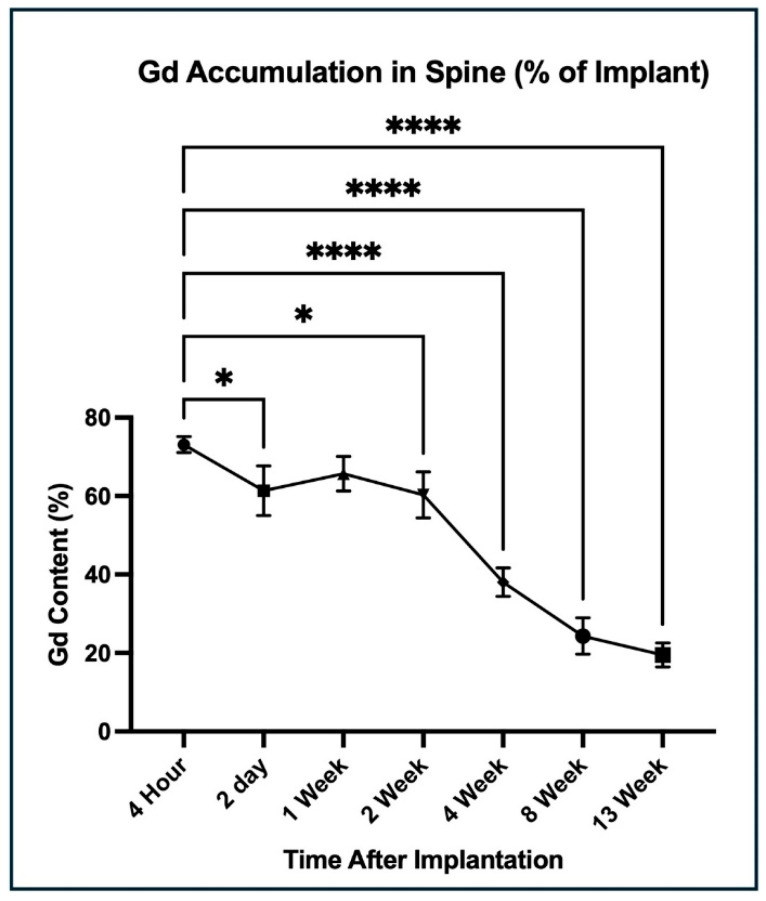
Time-course of Gd content in the spine. Points show mean Gd % detected by ICP-MS (*n* = 3), with error bars showing SD. Immediately following implantation, Gd content fell dramatically. Between 1 week and 8 weeks, Gd content declined gradually. Gd content was 19.5% at 13 weeks. * = *p* ≤ 0.05, **** = *p* ≤ 0.0001.

**Figure 5:**
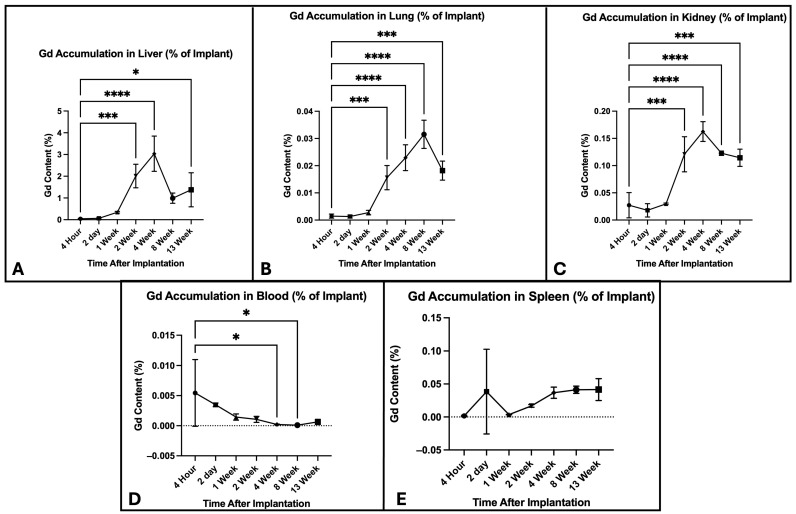
Time-course of Gd accumulation in blood and peripheral organs. The points show the mean Gd % detected by ICP-MS (*n* = 3), with error bars showing SD. Gd content was observed to increase in the liver, lung, and kidney until around weeks 4–8. The mean Gd percentage remained relatively stable throughout the study. * = *p* ≤0.05, *** = *p* ≤0.001, **** = *p* ≤0.0001.

Within 4 h of implantation, the average Gd content in the spine, including implants, was 73.1% of the pre-surgical implant content (Table S2, Figure 4). At 2 days, the average content fell further to 61.3%, and then remained relatively stable through the 2-week time point. From that point, Gd gradually declined to 19.5% at 13 weeks. Overall, a statistically significant down trend was observed in the mean Gd percentage compared to the baseline at 4 h (F(6,14) =67.53, *p* < 0.0001). Average Gd in the blood peaked 4 h post-operatively, with a value of 0.01% of the original implant (Table S2, Figure 5). Gd in the blood was <0.005% of starting content 2 days post-operatively and for the remainder of the study, with further down trending; however, the mean Gd percentages were not overall statistically significant compared with those at 4 h (F(6,14) = 2.62, *p* = 0.065). The liver exhibited the greatest amount of Gd accumulation among the peripheral organs. It reached an average maximum of 3.0% of the original concentration at 4 weeks, then declined to 1.4% by 13 weeks. The mean Gd was significantly increased, especially at 2 weeks and 4 weeks, compared with the baseline (F(6,14) = 16.01, *p* < 0.0001). Average Gd in the lungs gradually peaked at 0.03% at 8 weeks. It then declined slightly to 0.02% at 13 weeks, with overall statistical significance compared to at 4 h (F(6,14) = 35.81, *p* < 0.0001). Average Gd in the spleen increased at 2 days, with a value of 0.04%, and from there remained steady over the course of the 13-week study period, and the Gd percentage at each time point was similar to baseline (F(6,14) = 1.497, *p* = 0.391). Average Gd in the kidney reached its greatest value of 0.16% at 4 weeks, with a decline to 0.11% at 13 weeks; this increase in Gd percentage was statistically significant compared to baseline (F(6,14) = 30.82, *p* < 0.0001). Additional details for the ANOVA summary are provided in Supplemental Table S3.

## 4. Discussion

This study aimed to understand the degradation rate and biodistribution of a novel peptide-based supramolecular polymer implant when deployed in a rat spinal fusion model using Gd-labeled material. We observed robust fusion at 10 weeks post-implantation consistent with our prior investigation of the SPS material, indicating that the Gd-tag did not inhibit the bioactivity of the implant [19]. For this reason, additional animals for palpation, imaging and histology to prove fusion were not utilized. Prior studies in rodents indicate that Gd tags attached to PA scaffolds similar to those in the present study have a half-life of 13.5 days when implanted into muscle tissue, with approximately 30% Gd remaining after 24 days [22]. The present study differs in that we examine the PA degradation profile when the labeled PA scaffold is implanted in the spine fusion setting and incorporated into newly formed bone tissue, and our findings indicate a relatively slower process (38% remaining after 28 days). At 13 weeks, ICP-MS indicates that 19.5% of the initial Gd remains in the spine. Given the otherwise robust degradation profile that we have seen prior to bone ossification, we believe the residual Gd signal enhancement likely represents Gd-DOTA or Gd-peptide fragment incorporation after degradation [25,26]. MRI revealed a notable loss of signal enhancement at the 8-week time point and complete loss of signal enhancement at the 13-week time point, which is consistent with prior studies that found MRI to have more limited sensitivity for detecting Gd-labeled PA when compared to ICP-MS [22].

We suspect that the longer degradation time noted herein is in large part related to Gd incorporation into newly formed bone [27]. A similar rodent study demonstrated the degradation rate of a magnesium-Gd alloy pin implant with fragmentation of the pin at 12 weeks and particle spread into the intramedullary cavity and cortical bone. In some cases, Gd remained in the bone up to 36 weeks post-implantation [28]. This observation supports the hypothesis that as the implant degradation rate and new bone formation rate converge, free Gd and Gd-labeled complexes may become trapped or incorporated into the bone at a low enough concentration or far enough from water molecules to avoid detection on MRI. Furthermore, as unbound Gd has been found to undergo rapid tissue clearance within 2 h [22], the signal we detected at 13 weeks via ICP-MS could represent bound Gd-labeled scaffold or more likely, Gd incorporated directly into new bone after degradation. Gd has been shown to be capable of integration into bone structures in vivo and into hydroxyapatite crystals in both human and animal models with very long half-lives [27,29,30]. Histological slices of the fusion mass support this hypothesis, as we observed normal bone tissue throughout the implant site without evidence of the SPS scaffold.

Gd accumulation in liver, blood, kidney, spleen, and lung tissue was assessed with ICP-MS to understand implant degradation product biodistribution, and ultimately, there was no meaningful accumulation of Gd in any peripheral organ. The blood, kidney, spleen, and lung showed no functional accumulation, as less than 0.2% of the total Gd implanted was detected for the duration of the study. The liver was the only exception, where a mild uptick in Gd concentration was noted at 4 weeks, peaking at 3% of the total Gd implanted, but then declined to 1% at 13 weeks. Additionally, the consistently low concentrations of Gd in the blood indicate adequate clearance rates (Figure 4 and Figure 5). Overall, these data show negligible accumulation of Gd-PA in peripheral organs and indicates that PA scaffold degradation products are primarily processed by the liver.

Similar distribution has been reported in other studies involving Sprague–Dawley rats. Myrissa et al. reported significant Gd accumulation in the spleen, lung, liver, and kidney following transcortical implantation of a magnesium-Gd alloy pin [28]. In that study, it was apparent that the spleen played a significant role within the first days after implantation, followed by the liver and kidney, while other tissues maintained low Gd levels [28]. This may become clinically relevant in patients who have impaired liver, spleen, or kidney function; in these patients, degradation product clearance could vary. However, it should be noted that in the study by Myrissa et al., these organs increasingly accumulated Gd over 36 weeks [28], whereas in the current study, the signal remained consistently low or peaked and then declined. This indicates a difference in accumulation and clearance of degradation products between Gd-labeled PA and magnesium-Gd alloy. The evidence presented suggests that Gd-PA degradation products are more effectively cleared from the body than magnesium-Gd alloy products.

Ongoing research is warranted in preparation for human clinical studies on these novel implant materials. Efficacy testing in large animal trials is currently underway in a sheep model of lumbar interbody fusion. Investigational device exemption-enabling studies are required to examine implant safety and toxicology. Also, as the present study demonstrated some remaining Gd at the 13-week time point, investigations over a longer time period (6–9 months) in a larger animal model utilizing implants akin to the full-sized clinical product may be necessary to assess the biodistribution of implant degradation products after full bone remodeling has taken place.

This study has several limitations. Individual differences in rat anatomy and surgeon technique may have allowed variations in implant placement and tissue harvesting. Moreover, this investigation is unable to confirm that the Gd label remained attached to the PA structure for the length of the study, truly representing PA clearance rather than merely Gd clearance. As unbound Gd chelate is rapidly cleared, Gd content as detected on ICP-MS may thus underestimate the amount of PA remaining in tissues. On the other hand, long-term retention of Gd in the bone tissue may represent direct bony incorporation of free Gd, as can be seen clinically [27,29], leading to an artificially high estimate of the potential PA remaining in the newly formed bone.

## 5. Conclusions

These results reveal important insights into the supramolecular polymer slurry degradation rate and biodistribution of degradation products. Thirteen weeks post-operatively, the Gd signal enhancement originating from the labeled scaffold plateaued at 19.5% of the original implanted mass within the fusion site, which likely represents the incorporation of released free Gd or Gd-PA fragments into new bone. Gd signal accumulation in peripheral organs was negligible, with a peak of 3% of the implanted mass detected in the liver 4 weeks post-operatively, followed by a decline. These findings suggest that degradation products of the peptide-based scaffold may be primarily cleared hepatically, with a clearance rate sufficient to prevent their accumulation.

## Figures and Tables

**Table 1:** Diagrammatic summary of animal numbers, time points, and outcome measures. Number of animals undergoing MRI and/or ICP-MS at each post-operative time point. White rats represent living animals while black rats represent those sacrificed at each time point. In vivo MRI was performed over the duration of the experiment on the same 3 animals that were sacrificed in week 13. Ex vivo MRI was performed on the 3 animals being sacrificed for ICP-MS at each time point. ICP-MS necessitated euthanasia—live animals remaining at each time point are tallied in the bottom row.

Time Point	4 h	2 Days	1 Week	2 Weeks	4 Weeks	8 Weeks	10 Weeks *	13 Weeks
in vivo MRI		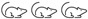	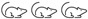	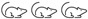	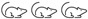	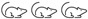		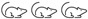
ICP-MS & ex vivo MRI	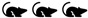	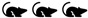	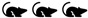	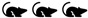	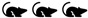	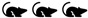		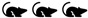
Remaining Animal Census	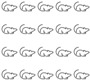	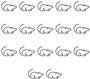	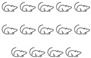	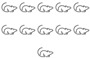	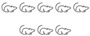	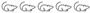	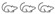	

* At 10 weeks, 2 animals were euthanized, and their spines were harvested for manual palpation to assess fusion and processed for histology. These animals did not undergo MRI or ICP-MS.

## Data Availability

Please contact the corresponding author for any data requests.

## Supplementary Materials

The following supporting information can be downloaded at https://www.mdpi.com/article/10.3390/jfb17030107/s1, Figure S1: Chemical analysis of PA molecules used in this study; Figure S2: Representative ex vivo axial T1 maps and T2 maps; Figure S3: Histologic evaluation of fusion mass 10 weeks post-operatively; Table S1: Scan parameters for in vivo MR imaging; Table S2: Biodistribution of Gd-PA implants using ICP-MS. Table S3: ANOVA analysis of ICP-MS signal over time from blood, spine, and organs.
