# Supplementary material for: Biodistribution and Biodegradation of an Osteoinductive Supramolecular Polymer Implant in a Rat Spinal Fusion Model
Source: J Funct Biomater. 2026 Feb 24;17(3):107. doi: 10.3390/jfb17030107 (PMC13027388; doi:10.3390/jfb17030107)
Supplement: Supplementary file 1 [file jfb-17-00107-s001.zip › jfb-4091837-supplementary.pdf]

Supplementary Material

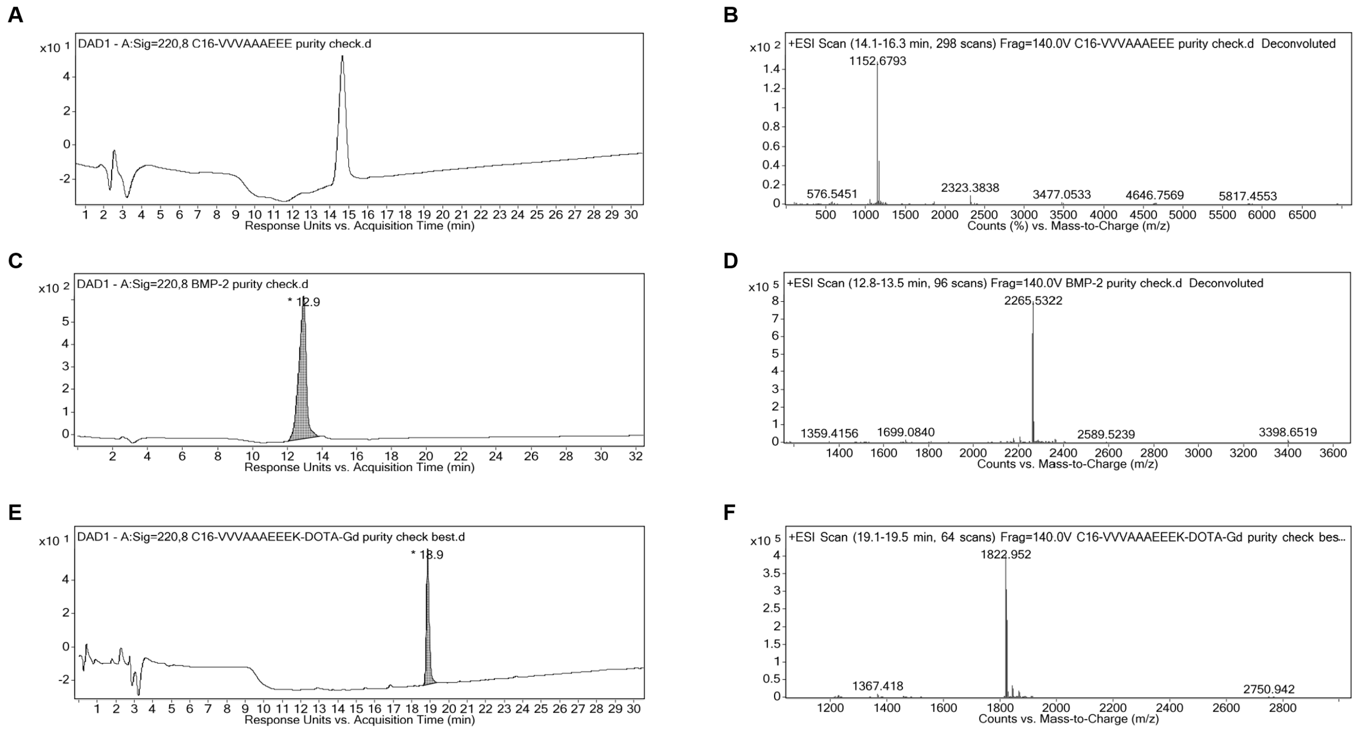

Figure S1: Chemical analysis of PA molecules used in this study. LC-MS traces of (A, B) diluent PA, (C, D) BMP-2-binding PA, and (E, F) Gd PA.

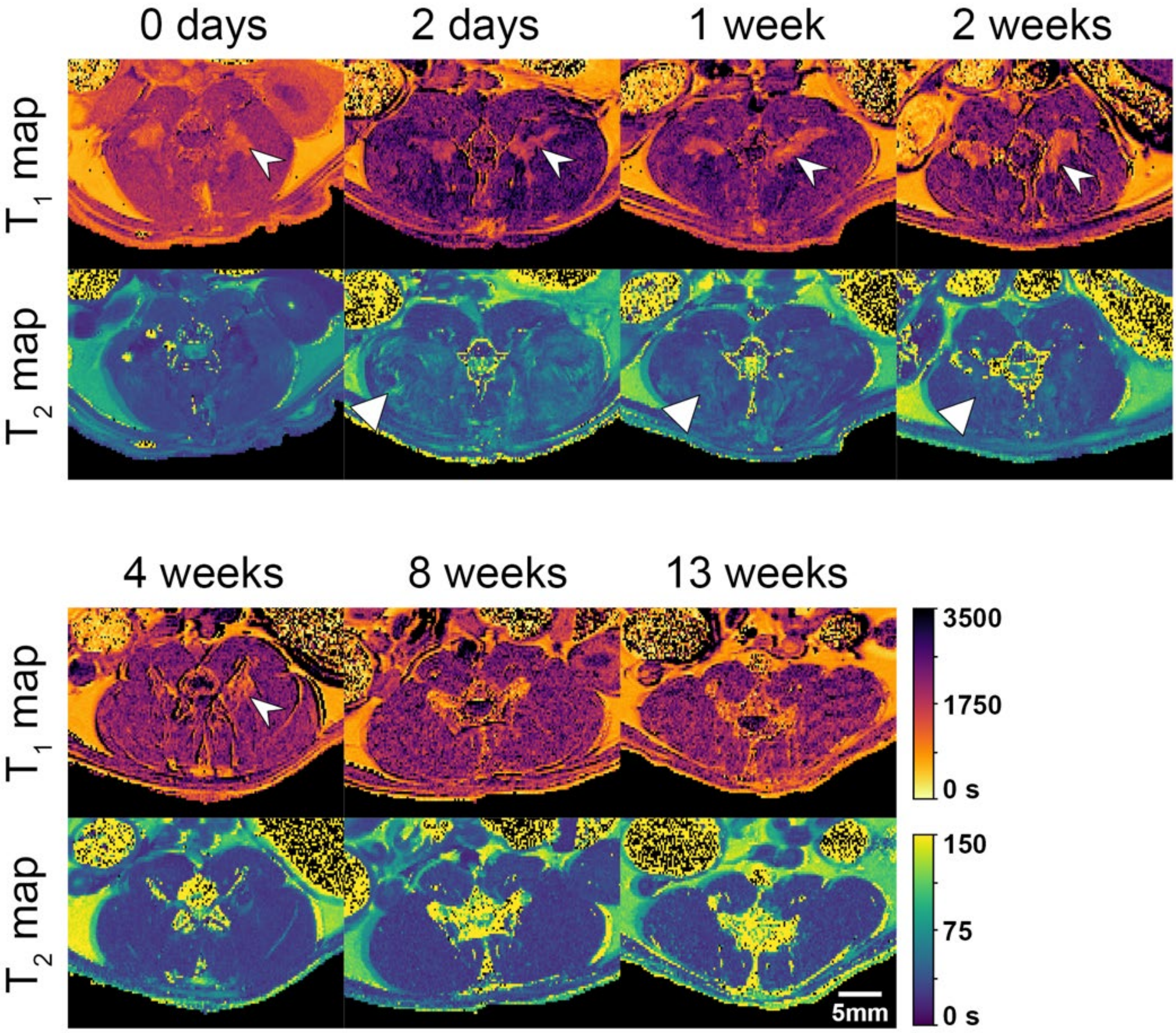

Figure S2: Representative ex vivo axial T1 maps (top row) and T2 maps (bottom row). Images are from a different animal at each time point. The 0-day image was collected several hours after euthanasia; the remaining images were obtained immediately after in-magnet euthanasia. T1 shortening in the Gd-PA implant is observed at 0 days through 4 weeks (barbed arrowheads). Diffuse postsurgical edema is significantly reduced by 2 weeks and fully resolved by 4 weeks (triangular arrowheads). The paired maps confirm that regions of signal enhancement on T1-weighted images are the result of T1 shortening due to Gd-PA and not affected by bleed-through of T2 signal from diffuse postoperative edema.

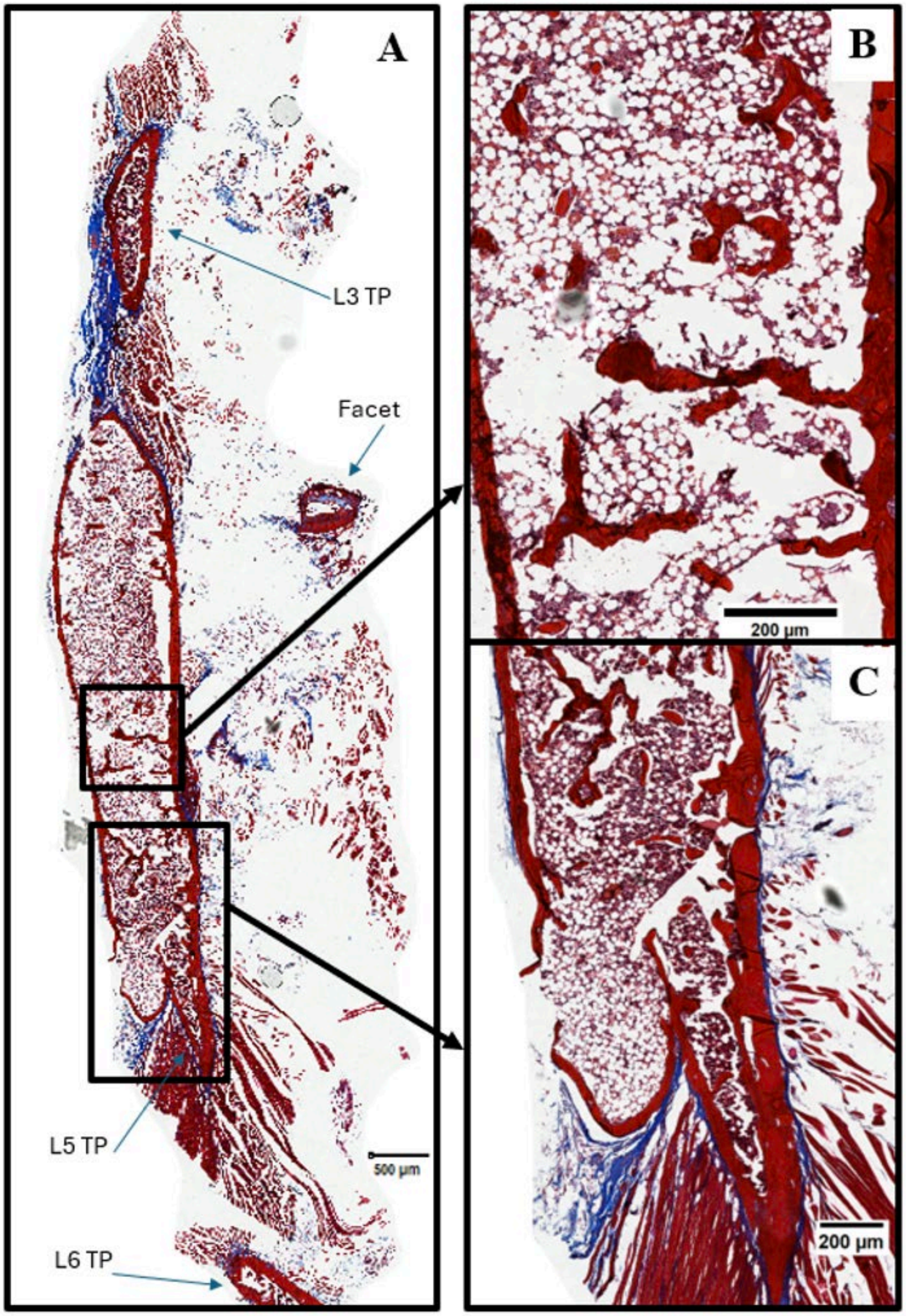

Figure S3: Histologic evaluation of fusion mass at 10-weeks post-op. (A) Representative Masson's trichrome histology thin section of an L4-L5 posterolateral fusion mass. (B) Between the L4 and L5 transverse processes (TPs), the collagen/peptide scaffold appears to be degraded and converted into bony fusion mass. Newly formed trabeculae can be visualized. (C) The fusion can be visualized directly superior to the L5 transverse process.

Table S1: Scan Parameters for in vivo MR imaging

	TR (ms)	TE (ms)	Flip angle	RARE factor	Matrix	Signal averages
**T_1-RARE**	750	5.31		4	256 x 256	8
**T_1-FLASH**	200	3.33	30	-	512 x 512	3
**T_2-RARE**	4000	31.5		8	256 x 256	2

Table S2: Biodistribution of Gd-PA implants using ICP-MS. Time-course of Gd decay in organs. Gd decay is calculated as percentage of Gd measured in the scaffold prior to implantation. Values are mean Gd % detected by ICP-MS.

Time after Surgery	4hr	2d	1wk	2wk	4wk	8wk	13wk
Spine	73.11	61.34	65.73	60.29	38.06	24.31	19.50
Blood	0.01	0.00	0.00	0.00	0.00	0.00	0.00
Kidney	0.03	0.02	0.03	0.12	0.16	0.12	0.11
Liver	0.04	0.06	0.34	2.01	3.04	0.99	1.38
Lung	0.00	0.00	0.00	0.02	0.02	0.03	0.02
Spleen	0.00	0.04	0.00	0.02	0.04	0.04	0.04

Table S3: ANOVA analysis of ICP-MS signal over time from blood, spine, and organs

Organ	Source	SS	df	MS	F	P-value	Effect Size (R^2)
**Blood**	Time	7.08E-05	6	1.18E-05	2.618	0.0647	0.5287
	Error	6.31E-05	14	4.51E-06			
**Kidney**	Time	0.06144	6	0.01024	30.82	P<0.0001	0.9296
	Error	0.004651	14	0.000332			
**Liver**	Time	22.27	6	3.712	16.01	P<0.0001	0.8728
	Error	3.246	14	0.2318			
**Lung**	Time	0.002549	6	0.000425	35.81	P<0.0001	0.9388
	Error	0.000166	14	1.19E-05			
**Spleen**	Time	0.005772	6	0.000962	1.497	0.2494	0.3908
	Error	0.008996	14	0.000643			
**Spine**	Time	8222	6	1370	67.53	P<0.0001	0.9666
	Error	284.1	14	20.29			
